# Research on Recognition of Motor Imagination Based on Connectivity Features of Brain Functional Network

**DOI:** 10.1155/2021/6655430

**Published:** 2021-02-12

**Authors:** Zhizeng Luo, Ronghang Jin, Hongfei Shi, Xianju Lu

**Affiliations:** ^1^Institute of Intelligent Control and Robotics, Hangzhou Dizanzi University, Hangzhou, China; ^2^The Fourth Affiliated Hospital, Zhejiang University School of Medicine, Hangzhou, China

## Abstract

Feature extraction is essential for classifying different motor imagery (MI) tasks in a brain-computer interface. To improve classification accuracy, we propose a novel feature extraction method in which the connectivity increment rate (CIR) of the brain function network (BFN) is extracted. First, the BFN is constructed on the basis of the threshold matrix of the Pearson correlation coefficient of the mu rhythm among the channels. In addition, a weighted BFN is constructed and expressed by the sum of the existing edge weights to characterize the cerebral cortex activation degree in different movement patterns. Then, on the basis of the topological structures of seven mental tasks, three regional networks centered on the C3, C4, and Cz channels are constructed, which are consistent with correspondence between limb movement patterns and cerebral cortex in neurophysiology. Furthermore, the CIR of each regional functional network is calculated to form three-dimensional vectors. Finally, we use the support vector machine to learn a classifier for multiclass MI tasks. Experimental results show a significant improvement and demonstrate the success of the extracted feature CIR in dealing with MI classification. Specifically, the average classification performance reaches 88.67% which is higher than other competing methods, indicating that the extracted CIR is effective for MI classification.

## 1. Introduction

Motor imagery (MI) refers to a thinking activity in which one can imagine completing a specific movement without the help of limb movements. MI can activate the motor neurons and network connections of damaged regions to some extent and even generate new neural compensation functions [1]. Researchers have sufficiently demonstrated that MI can activate the motor-related cortex as effectively as actual motion [2–4]. MI can be divided into simple imagery, which involves a single part of limb movements, and compound imagery, which involves not less than two parts of limb movements [5, 6]. With the advancement of brain science in recent years, MI training has gradually become a new rehabilitation method for patients with limb motor dysfunction caused by brain injury [7–9].

The functions of different brain regions are specific, and different limbs correspond to different sensorimotor regions of the cortex [10]. Simple limb MI involving a single limb can activate specific regions corresponding to limb parts. As early as the year 2000, Carlo et al. [3] found that when left- or right-hand movement is imagined, the contralateral brain functional region is activated. Compound limb MI allows multiple functional regions to participate synergistically, which is conducive to activating the damaged motor neurons in patients with motor dysfunction [5, 11]. However, most research focused on analyzing the changes in electroencephalogram (EEG) signals caused by simple limb MI. Currently, less work is reported about compound limb MI. [12] analyzed the feature differences between simple and compound limb MIs in event-related desynchronization (ERD)/event-related synchronization (ERS) and found that the ERD of compound limb MI is more intense. In addition, they conducted a separability study on simple and compound limb MIs. Multiclass MI, especially compound limb MI, is more realistic and instructive for patient rehabilitation training. Moreover, the evolution from simple to compound limb MI expands the number of identifiable MI patterns and improves the ability of brain-computer interface (BCI) to control external devices.

The ERD/ERS phenomenon is caused by the resonance of numerous neurons on physiological electrical signals, indicating the interaction between neurons and local neurons in a certain frequency band EEG, which mainly appears on the sensory motion region corresponding to electrodes C3, C4, and Cz. For instance, the ERD on the left side of the motor region (C3) is observed in right-hand MI, whereas that on the right side of the motor region (C4) is observed in left-hand MI. For foot MI, the ERD is in the midline central region (Cz) [12, 13]. ERD is related to the mu (8–12 Hz) and beta (13–30 Hz) rhythms of EEG signals. However, some frequencies in the beta rhythm are the harmonic waves of the mu rhythm. Thus, mu rhythm is associated with motion or MI. The ERD in the mu rhythm can be used as a direct indicator to reflect the excitation degree of neurons in the cortical region and assess neurodevelopment [14, 15]. [13] used the ERD values on electrodes C3 and C4 to characterize the left- and right-hand MIs, respectively. [16] calculated the ERD in the mu rhythm to reflect sensorimotor cortex activation and evaluate cortex excitability during MI. The ERD calculation is based on the signals of the brain region under test, whereas compound limb MI requires the collaborative participation of multiple brain regions. Given that each brain region does not operate independently, the cortical activity differences between simple and compound limb MIs can only be analyzed from the ERD.

The brain function network (BFN) is based on the complex network theory, which describes the statistical functional connection relationship in various regions of the brain [17–20]. Furthermore, the BFN can construct brain functional topology and integrate the connectivity strength between different regions. In addition, the BFN not only reflects the global activity of the cerebral cortex but also provides a highly reliable performance for distinguishing multiclass MI. Many studies have demonstrated that the BFN is an effective method for describing the coordination among different brain regions. For example, [21] used local and global efficiency to analyze the relationship between brain information transmission efficiency and working memory performance in young and old people. [22] performed spectral decomposition on BFNs to classify 4-class tasks. However, the brain is a time-varying coupled chaotic nervous system. Under different classes of movements, the BFN dynamically changes, and the topological structure of the network varies. Furthermore, no absolute 0–1 relationship exists between nodes in the BFN, but a degree of connectivity does. Therefore, BFN features are caused by changes of network location and connection. By contrast, most studies on BFN ignored these points, resulting in insufficient network information.

To address these drawbacks, we propose a framework for multiclass MI classification based on the BFN theory. In our framework, a weighted BFN is constructed, and the sum of the existing edge weights is used to characterize the cerebral cortex activation degree in different MIs. The main advantage of the framework is that the novel feature connectivity increment rate (CIR) is a regional network feature centered on the functional cortex area, which reflects the changes of network location and connectivity and reduces the loss of network information. Thus, three regional networks centered on the C3, C4, and Cz channels are constructed by the BFN topological structures of seven mental tasks. The CIRs of these networks are used as features to distinguish MI classification via the support vector machine (SVM). In summary, this work contributes the following:

1. The construction of weighted BFNs facilitates the study of cerebral cortex activation degree in different movement patterns
2. The feature CIR can reflect the dynamic changes of the network and provides a new idea for feature extraction
3. The study of multiclass MI is conducive to expanding the instruction set of the BCI.

The rest of this paper is organized as follows: “Methods and Materials” discusses the construction of BFN and feature extraction method, as well as the experimental scheme. Experimental results are presented in “Results,” followed by the discussion and conclusions in “Discussion and Conclusions”.

## 2. Methods and Materials

### 2.1. Construction of BFN

The BFN describes the statistical functional connection relationship in various regions of the brain. It has small-world network attributes and belongs to the undirected network in complex networks [23]. EEG signals, functional magnetic resonance imaging, and magnetoencephalography are commonly used as signal sources for BFN construction. The steps are listed as follows:

(Step 1) We select the appropriate network nodes. In this study, given that multichannel EEG signals are used, we define each electrode on the scalp surface as a network node
(Step 2) We quantify the functional connectivity relationship between network nodes. One of the commonly used methods is Pearson's correlation coefficient [24]. The formula is shown as follows:

(1) $$\begin{array}{c} r_{ij}=\frac{\sum_{t=1}^{T}\left[x_{i}\left(t\right)-\bar{x_{i}}\right]\left[x_{j}\left(t\right)-\bar{x_{j}}\right]}{\sqrt{\sum_{t=1}^{T}{\left[x_{i}\left(t\right)-\bar{x_{i}}\right]}^{2}\sum_{t=1}^{T}{\left[x_{j}\left(t\right)-\bar{x_{j}}\right]}^{2}}}i,j=1,2,\cdots ,N, \end{array}$$

where *x*_*i*_(*t*) and *x*_*j*_(*t*) are the sampling values of nodes *i* and *j* at time *t*, respectively; $\bar{x_{i}}$ and $\bar{x_{j}}$ are the average sampling values of nodes *i* and *j*, respectively; and *N* is the number of network nodes. We can obtain a *N* × *N* connection coefficient symmetry matrix. In this case, the network is a weighted network, and *r*_*ij*_ is the weight. A higher correlation coefficient is proportional to a higher linear relationship

(Step 3) We obtain BFN by thresholding the weighted network. By selecting an appropriate threshold value (*δ*) and performing threshold processing on the connection coefficient matrix, we obtain a 0–1 adjacency matrix, which can be expressed as follows:

(2) $$\begin{array}{c} a_{ij}=\left\{\begin{array}{c} \begin{array}{cc} 1 & \left|r_{ij}\right|\geq \delta \end{array}\\ \begin{array}{cc} 0 & \left|r_{ij}\right|<\delta \end{array} \end{array}\right.. \end{array}$$

*a*_*ij*_ = 1 indicates that a connection edge exists, and the correlation between nodes *i* and *j* is strong, which otherwise does not exist and is weak, respectively. In accordance with the adjacency matrix, the topological structure of the BFN can be obtained.

### 2.2. Feature Extraction Based on BFN

#### 2.2.1. Motivation

**Figure 1:**
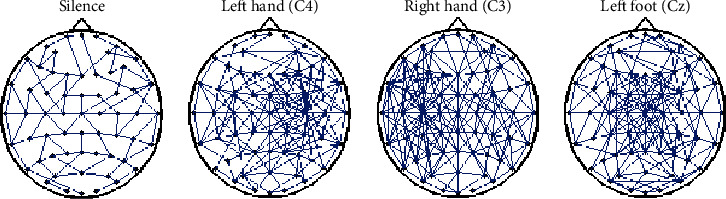
BFN topological structures of simple limb MI. Note: Contents in parentheses represent the regions with the highest degree of aggregation corresponding to the current MI.

Neurophysiological studies have shown that the MI for different limb movements activates the corresponding limb motor regions in the cerebral cortex [25]. In “Methods and Materials,” we obtain the BFN topological structures of a simple limb MI.  Figure 1 intuitively shows that when imagining the left-hand motion, the network in the vicinity of electrode C4 in the corresponding motor control cortex region is highly agglomerated. Similarly, for the right-hand and left-foot MI, the network in the vicinity of electrodes C3 and Cz in the corresponding motor control cortex region is highly agglomerated, respectively. No clustering trend exists in the silent state network. Electrodes C3 and C4 correspond to the hand control region of the cerebral cortex, and electrode Cz corresponds to the foot control region of the cerebral cortex. Furthermore, in different movement patterns, the number of connections between different regions vary, indicating the varying cerebral cortex activation degree. In addition, after threshold processing, only a connected or unconnected state exists between the BFN nodes, and many useful information will be lost. The functional connection between nodes is not absolute but has a degree of connectivity. Therefore, on the basis of network thresholding, the connection coefficient is used as a weight to redescribe the functional connectivity relationship between network nodes. At this time, the BFN is called the weighted BFN. In this study, we use the sum of the existing edge weights as the function network connectivity value (*C*) to characterize the cerebral cortex activation degree in different MIs.

#### 2.2.2. Feature Calculation

As mentioned above, the function network connectivity value (*C*) can be computed as

(3) $$\begin{array}{c} C=\underset{i,j\in V,i<j}{\sum }r_{ij}a_{ij}, \end{array}$$

where *r*_*ij*_ is the connection coefficient between network nodes *i* and *j*, *a*_*ij*_ is shown in Eq. (2), and *V* is the set of network nodes.

For normalization, we take the absolute value of the ratio of *C* during MI to that during the resting period before the beginning of imagination as the CIR. The CIR is used as a novel feature for classifying multiclass MI and calculated as follows:

(4) $$\begin{array}{c} \text{CIR}=\left|\frac{C_{\text{image}}}{\bar{C_{\text{rest}}}}\right|, \end{array}$$

where $\bar{C_{\text{rest}}}$ is the average of *C*_rest_ of all subjects in the silent state.

#### 2.2.3. Threshold Selection

The selection of the threshold value plays a vital role in the study of brain function network characteristics. When the value of the threshold value is selected too large, the edge of the network will decrease, and the network sparsity will increase, resulting in the loss of network information. If the threshold is too small, a large number of weakly correlated edges will be introduced, which will increase the density of the network and increase the complexity of the brain network, which is not conducive to the analysis of effective brain information. So far, there is no consistent conclusion on the selection of brain function network threshold. The principles for selecting the threshold in this paper are as follows: (1) The brain function network should have significant small-world network characteristics. (2) The brain function network should have significant connectivity.

**Figure 2:**
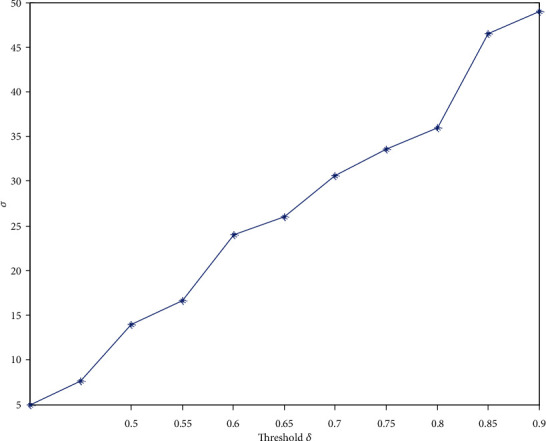
Characteristic diagram of comprehensive index changing with threshold.

Current research shows that the brain function network has been proven to have the characteristics of a small-world network. The formula for calculating the characteristics of the small-world network is as follows:

(5) $$\begin{array}{c} \gamma =\frac{C_{\text{real}}}{C_{\mathrm{rand}}}\gg 1, \end{array}$$

(6) $$\begin{array}{c} \lambda =\frac{L_{\text{real}}}{L_{\mathrm{rand}}}\approx 1, \end{array}$$

(7) $$\begin{array}{c} \sigma =\frac{\gamma }{\lambda }, \end{array}$$

where *C*_real_ and *C*_rand_ are the average clustering coefficients of real brain function network and random network of the same node size. *L*_real_ and *L*_rand_ are the average path lengths of real brain function network and random network of the same node size. *σ* is a comprehensive index to measure whether the network has the characteristics of a small-world network. When *σ* > 1, it indicates that the network has small-world attributes; otherwise, it does not, and the strength of small-world attributes is positively correlated with the size of *σ*. Different subjects have different thresholds for motor imagination when performing different actions. The article takes one of the subjects performing the left-hand motor imagination task as an example.  Figure 2 is a characteristic diagram of comprehensive index changing with threshold.

It can be seen from Figure 2 that the size relationship between comprehensive index *σ* and threshold *δ* is positively correlated. However, the number of edges connected to nodes in the network will decrease with the increase of threshold *δ*, and the average node degree *K* of the network will decrease. When the threshold *δ* is too large, there will be more isolated nodes in the network, resulting in the decline of the functional connectivity of the network, and thus, the integrity of the brain network information cannot be guaranteed. Therefore, the average node degree *K* of network cannot be less than the natural logarithm of the number of network nodes *N*, that is, *K* ≥ ln*N* = ln(60) ≈ 4.09. In this paper, the specific steps to determine the threshold are as follows:

1. When *δ* = 0.85, the value range of the average node degree of the brain network is 4~18 and meets the requirements *K* ≥ ln*N*
2. When the threshold is superimposed to 0.9 according to the step length of 0.05, the average node degree *K* of the network decreases due to the increase of threshold *δ*. At this time, the condition *K* ≥ ln*N* is not satisfied
3. When *δ* = 0.8, although the average node degree *K* meets the requirements under this threshold, the comprehensive index *σ* is smaller than the value when *δ* = 0.85.

Therefore, this paper chooses the threshold *δ* = 0.85, and the brain function network corresponding to this threshold also guarantees significant small-world characteristics and functional connectivity. The selection of thresholds for different motor imagination of all subjects is shown in Table 1.

#### 2.2.4. Proposed Algorithm

To illustrate the feature extraction method clearly, we implement the algorithm flow as follows:

(Step 1) According to different mental tasks, we construct the corresponding BFNs
(Step 2) Based on the corresponding relationship between the motor control cortex and the MI movements, we divide the threshold nodes into regions reasonably with the help of the BFN topological structures
(Step 3) To quantify the functional connectivity between network nodes in each region, we use the Pearson correlation coefficient
(Step 4) Calculate *C*_image_ during MI and $\bar{C_{\text{rest}}}$ during rest
(Step 5) Calculate the CIR of each region.

### 2.3. Experimental Scheme

**Figure 3:**
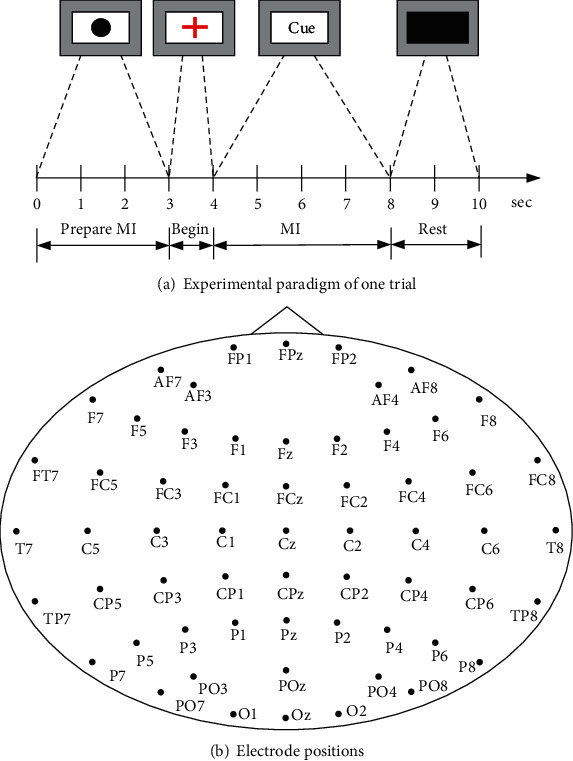
Experimental paradigm and electrode positions.

We designed seven classes of mental tasks using prompt words, such as “Left Hand,” “Right Hand,” “Left Foot,” “Left Hand + Left Foot,” “Right Hand + Left Foot,” “Left Hand + Right Hand,” and “Silence,” to express three tasks of simple limb MI, three tasks of compound limb MI, and the silent state, respectively. The silent state was used for comparison. The implementation sequence of the specific experimental mode is shown in Figure 3. The data acquisition time of each trial was 10 s. From 0–3 s, when the screen displayed a black dot, the subject was in the preparation stage of MI. From 3–4 s, the black dot on the screen turned into a red cross to remind the subject that mental tasks were about to begin. From 4–8 s, the screen displayed the character indication (“Right Hand,” “Right Hand + Left Foot,” and so on). The subject had to concentrate on performing the corresponding movement kinesthetically according to the screen and avoid any action during MI. From 8–10 s, the subject entered the rest stage when the screen was all black.

The subjects included twelve healthy students (8 males and 4 females, 21-25 years old) with no history of brain diseases. They were right-handed and had no prior experience with MI. They signed informed consent. The trial was conducted under the condition that the subjects were conscious and well rested. The subjects performed MI 10 times in each mental task, with a 5 s rest between two trials. The subjects rested for 5 mins before the next mental task. A total of 840 groups of EEG signals were recorded in the trials. One group of EEG signals was a multichannel data recorded by a subject in trial (a complete preparation stage, prompting stage, imagination stage, and rest stage).

In the process of motor-imaging EEG signal acquisition, blinking is unavoidable. Electrooculogram (EOG) signals caused by eyeball or eyelid movements propagate along the skull and merge with EEG signals, causing EEG artifacts. In order to filter out ocular artifacts, in each round of experiment, the EEG acquisition device needs to continuously and synchronously collect the subject's EEG and additional independent EOG signals, so that EEG preprocessing can eliminate artifacts.

In this study, the EEG signals were recorded by an EEG- amplifier of Neuracle, and the Ag/AgCl scalp electrodes were referenced to the A1 and A2 electrodes as ground. The EEG signals were recorded at the following 60 positions of the international 10-20 system: Fp1, Fpz, Fp2, AF7, AF3, AF4, AF8, F7, F5, F3, F1, Fz, F2, F4, F6, F8, FT7, FC5, FC3, FC1, Fcz, FC2, FC4, FC6, FT8, T7, C5, C3, C1, Cz, C2, C4, C6, T8, TP7, CP5, CP3, CP1, Cpz, CP2, CP4, CP6, TP8, P7, P5, P3, P1, Pz, P2, P4, P6, P8, PO7, PO3, Poz, PO4, PO8, O1, Oz, and O2. The sampling rate of the amplifier was 250 Hz, and the notch filter was 50 Hz. Moreover, the band-pass filtering range was 0.1–100 Hz. Besides, fast independent component analysis was used in the preprocessing.

## 3. Results

### 3.1. BFN in Different Movements

**Figure 4:**
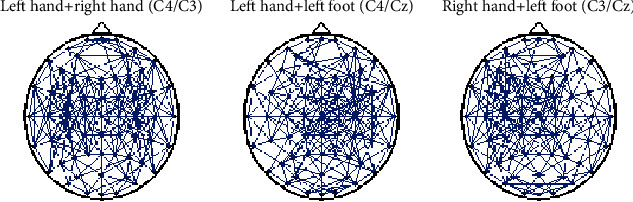
BFN topological structures of compound limb MI.

We constructed a BFN based on the denoised experimental data. First, we performed an 8–13 Hz band-pass filter on the signals of each channel and then extracted the mu rhythm. Second, we calculated the Pearson correlation coefficients between the mu rhythms of any two channel signals and obtained a60 × 60 connection coefficient matrix. Finally, the matrix was processed by the threshold. We selected threshold *δ* = 0.85 and obtained a 0–1 adjacency matrix.  Figure 1 shows the BFN topological structures of simple limb MI, whereas Figure 4 shows the BFN topological structures of compound limb MI.

When imagining left-/right-hand compound motion, the network in the vicinity of electrode C4/C3 in the corresponding motor control cortex region is highly agglomerated. Similarly, for the left-hand/-foot and right-hand/left-foot MI, the network in the vicinity of electrodes C4/Cz and C3/Cz in the corresponding motor control cortex region is highly agglomerated. Combining Figures 1 and 4, we observe that different movement patterns result in different partitions. Therefore, based on the threshold network, we constructed three weighted BFNs with electrodes C3, C4, and Cz as the center, which were called the C3, C4, and Cz region BFNs. Then, we calculated the CIR of these three BFNs and composed the three-dimensional feature vectors [*CIR*_*C*3_, *CIR*_*C*4_, *CIR*_*Cz*_] to characterize EEG signals.

**Figure 5:**
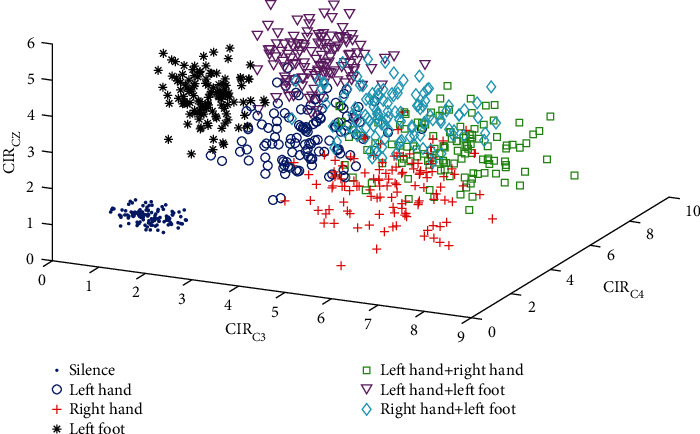
CIR distribution of seven mental tasks.

The C3 region BFN was constructed as follows: We took electrode C3 as the center. Then, we selected appropriate electrodes to reduce redundant information based on the electrode distribution relationship of the motor control cortical region that right-hand MI corresponds to. Correspondingly, we used electrodes F1, F3, F5, FC1, FC3, FC5, C1, C3, C5, CP1, CP3, CP5, P1, P3, and P5 as the network nodes. Similarly, electrodes F2, F4, F6, FC2, FC4, FC6, C2, C4, C6, CP2, CP4, CP6, P2, P4, and P6 were used as the network nodes of the C4 region BFN. Electrodes F1, F2, Fz, FC1, FC2, FCz, C1, C2, Cz, CP1, CP2, CPz, P1, P2, and Pz were used as the network nodes of the Cz region BFN. We separately calculated the CIR in the C3, C4, and Cz regions of the 840 groups of EEG signals recorded in the experiment, which were composed of CIRs.  Figure 5 shows the distribution of the CIRs in seven movement patterns. CIR_*C*3_ represents the C3 region CIR. The mean and standard deviation of the CIRs for seven mental tasks are shown in Table 2.

Figure 5 intuitively shows that the feature vector [*CIR*_*C*3_, *CIR*_*C*4_, *CIR*_*Cz*_] can distinguish the EEG signals of seven mental tasks. Especially, the data points of the silence and the left-foot movement are highly distinguished from the other movement patterns. Although overlaps among the data points of movements (except for silence and left foot) are observed, the degree of distinction is slightly evident. Figure 4 and Table 1 indicate that among the three features of *CIR*_*C*3_, *CIR*_*C*4_, and*CIR*_*Cz*_, the three eigenvalues of silence are approximately 1, which is theoretically consistent. The *CIR*_*C*4_ of the left-hand MI is larger than those of the other two. For right-hand and left-foot movements, similar conclusions are drawn. Additionally, for both hands, *CIR*_*C*3_ and *CIR*_*C*4_ are considerably larger than *CIR*_*Cz*_. The *CIR*_*C*4_ of the left-hand/foot MI is the largest, followed by *CIR*_*Cz*_. The *CIR*_*C*3_ of the right-hand/left-foot MI is the largest, followed by *CIR*_*Cz*_. These results are consistent with the division of the limb motor control function in the cerebral cortex. The CIR distribution of 7 mental tasks are be further plotted in each individual subject (see Figure S1 for details).

### 3.2. Classification

To evaluate the significant difference among the seven mental tasks of each subject, the article pairs different mental tasks in pairs. 21 comparison groups were constructed according to 7 groups of tasks, and the differences of the feature vector [*CIR*_*C*3_, *CIR*_*C*4_, *CIR*_*Cz*_] among each comparison group were analyzed by the multifactor analysis of variance. The comparison results are shown in Table 3. This result reveals that there are significant differences in these mental tasks.

To evaluate our method, we chose SVM to classify seven mental tasks. To evaluate the classification performance of feature vector, the 5-fold cross-validation technique was selected. To train a multiclass classifier, we used the one-versus-rest strategy. We randomly divided each MI task data set into 5 parts and took turns to take 4 parts as training set and 1 part as testing set. Since there were seven kinds of MI tasks, there were 28 sets of samples were used as the training set, and 7 sets of samples were used as the testing set. In order to make the classification results more accurate, this process was repeated 10 times.

Table 4 shows that the average classification accuracy of the CIR in each mental task. Except for silence, the highest accuracy reaches 91.88%. The average accuracy of the CIR for seven mental tasks is 86.44%. This result indicates that our proposed feature extraction method is promising for the BCI research.

Besides, for motor imagery-based BCI system, a large number of algorithms have been applied to extract EEG features of different mental tasks such as band power (BP), autoregressive (AR), power spectral density (PSD), and common spatial patterns (CSP) [26–29]. However, the above feature extraction method is widely used only in the pattern recognition of 2-class mental tasks, and there is a clear deficiency in the recognition of multiclass MI [30]. CSP is a method for multichannel spatial filtering of binary data and has become the mainstream method of EEG signal processing. Since the nature of CSP is a binary case, the literature [12] uses a one-versus-rest scheme to modify the CSP algorithm and proposes multiclass CSP (multi-CSP), multiclass CSP based on generalized eigenvector (multi-GECSP), and multiclass stationary Tikhonov-regularized CSP (multi-sTRCSP) to solve multiclass MI classification. The classification results are satisfactory. The interested readers for detailed information should refer to the literature [12]. In order to better highlight the effectiveness of CIR, in this study, we use multi-CSP, multi-GECSP, and multi-sTRCSP algorithms to extract features and classify and compare the results with those of CIR, as shown in Table 5.

*t*-test was used to analyze the difference between the proposed method and other methods, and the results show that the proposed method is significantly different from the multi-CSP (*p* < 0.01) and multi-GECSP (*p* < 0.01) but not significantly different from the multi-sTRCSP (*p* = 0.065). Although the proposed method and the multi-sTRCSP are not significantly different, the classification accuracy of CIR proposed in this study is higher than that of multi-sTRCSP, which exceeds 2.37% gain. Therefore, we can draw a conclusion that the CIR can use an MI feature to improve the classification performance.

## 4. Discussion and Conclusions

In this study, we proposed CIR as a novel BFN feature and applied it to multiclass MI classification research. The CIR was proposed for the dynamic changes of BFN connection characteristics under different movement patterns to reflect the changes of network location and connection well. Additionally, the CIR was fully considered in the network topology features of single and compound limb MIs, thereby improving the effectiveness of network measurement. Compared with the traditional network measures, the CIR has the characteristic of a regional network centered on the functional cortex region, which reduces the loss of network information and includes more functional connectivity information among brain regions. At the same time, CIR compares with multi-CSP, multi-GECSP, and multi-sTRCSP algorithms in dealing with multiclass MI. The classification results verify that the multi-sTRCSP algorithm is superior to the multi-CSP and multi-GECSP. However, the classification accuracy of CIR proposed in this study is higher than that of multi-sTRCSP, which further demonstrates the effectiveness of CIR.

However, our method still has some drawbacks and deserves further study. (1) For the more complex movements in MI, the BFN has a denser topology and a more obvious degree of clustering. Further research can expand the functional regions appropriately and increase the dimensions of feature vector to improve classification performance. (2) Computational complexity is a concern for real-time BCI. Hence, a topic worth considering is to reduce the computational complexity of our method, which will achieve generalization in practical applications.

## Figures and Tables

**Table 1:** The selection of thresholds for different motor imagination of different subjects.

Task subject	S1	S2	S3	S4	S5	S6	S7	S8	S9	S10	S11	S12
S	0.85	0.80	0.85	0.75	0.85	0.80	0.80	0.85	0.80	0.85	0.80	0.85
LH	0.85	0.75	0.80	0.75	0.80	0.80	0.85	0.85	0.80	0.75	0.85	0.80
RH	0.80	0.80	0.80	0.70	0.80	0.85	0.80	0.80	0.80	0.80	0.80	0.75
LF	0.85	0.85	0.80	0.75	0.85	0.80	0.80	0.80	0.75	0.85	0.80	0.85
LH&RH	0.75	0.70	0.75	0.70	0.75	0.75	0.70	0.80	0.75	0.75	0.75	0.75
LH&LF	0.70	0.80	0.70	0.80	0.70	0.70	0.75	0.75	0.70	0.80	0.70	0.80
RH&LF	0.75	0.75	0.80	0.70	0.80	0.75	0.70	0.75	0.80	0.75	0.75	0.70

Notes: Silence is abbreviated as S, left hand is abbreviated as LH, right hand is abbreviated RH, left foot is abbreviated LF, left hand and right hand is abbreviated as LH&RH, left hand and left foot are abbreviated as LH&LF, and right hand and left foot are abbreviated as RH&LF.

**Table 2:** Mean and standard deviation of CIRs in seven mental tasks.

	CIR_*C*3_	CIR_*C*4_	CIR_*Cz*_
Silence	1.46 ± 0.13	1.28 ± 0.14	1.07 ± 0.19
Left Hand	3.11 ± 0.78	5.71 ± 0.96	2.02 ± 0.49
Right Hand	5.87 ± 0.94	3.22 ± 0.71	2.01 ± 0.63
Left Foot	2.32 ± 0.49	2.16 ± 0.49	4.22 ± 0.59
Left Hand + Right Hand	6.11 ± 0.99	6.21 ± 1.00	2.09 ± 0.59
Left Hand + Left Foot	3.24 ± 0.55	5.98 ± 0.87	4.21 ± 0.51
Right Hand + Left Foot	5.95 ± 0.87	3.42 ± 0.65	3.89 ± 0.57

**Table 3:** *p* values of the feature vector.

Task	[*CIR*_*C*3_, *CIR*_*C*4_, *CIR*_*Cz*_]						
	S	LH	RH	LF	LH&RH	LH&LF	RH&LF
S	≫0.05	<0.01	<0.01	<0.01	<0.01	<0.01	<0.01
LH	<0.01	≫0.05	0.013	<0.01	0.012	0.021	0.017
RH	<0.01	0.013	≫0.05	<0.01	0.015	<0.01	<0.01
LF	<0.01	<0.01	<0.01	≫0.05	<0.01	<0.01	<0.01
LH&RH	<0.01	0.012	0.015	<0.01	≫0.05	0.018	0.026
LH&LF	<0.01	0.021	<0.01	<0.01	0.018	≫0.05	<0.01
RH&LF	<0.01	0.017	<0.01	<0.01	0.026	<0.01	≫0.05

**Table 4:** Average classification accuracies for feature (%).

Testing set	[*CIR*_*C*3_, *CIR*_*C*4_, *CIR*_*Cz*_]						
	S	LH	RH	LF	LH&RH	LH&LF	RH&LF
Fold-1	98.60	86.95	88.60	90.55	83.56	82.56	82.63
Fold-2	97.56	81.37	85.67	88.56	86.68	80.89	85.59
Fold-3	96.80	85.49	81.02	91.88	84.23	79.55	84.54
Fold-4	97.90	82.56	82.54	88.15	80.79	85.23	79.16
Fold-5	95.42	82.89	84.56	86.96	83.21	86.01	86.72
Average	97.26 ± 1.21	83.85 ± 2.29	84.48 ± 2.92	89.22 ± 1.97	83.69 ± 2.11	82.84 ± 2.76	83.73 ± 2.96

**Table 5:** The average classification accuracy of the seven mental tasks under different methods of each subject.

Method	Subject												
	S1	S2	S3	S4	S5	S6	S7	S8	S9	S10	S11	S12	Mean
Multi-CSP	77.61	73.23	82.23	75.63	78.98	83.33	70.55	82.23	78.56	77.37	79.93	81.11	78.40
Multi-GECSP	77.64	70.23	79.28	81.13	69.95	75.56	76.79	77.21	72.89	75.32	78.21	73.59	75.65
Multi-sTRCSP	79.16	85.32	83.32	80.23	86.66	77.96	83.56	81.18	82.77	84.34	82.58	84.14	82.60
Proposed method	82.51	83.76	89.82	87.76	84.33	87.36	82.17	86.69	79.95	88.93	79.86	86.54	84.97

## Data Availability

All data included in this study are available upon request by contact with the corresponding author.

## Supplementary Materials

Supplementary Materials Figure S1: the CIR distribution of the 7 mental tasks in each individual subject.
