# Supplementary figures and images for: Research on Recognition of Motor Imagination Based on Connectivity Features of Brain Functional Network
Source: Neural Plast. 2021 Feb 12;2021:6655430. doi: 10.1155/2021/6655430 (PMC7895585; doi:10.1155/2021/6655430)

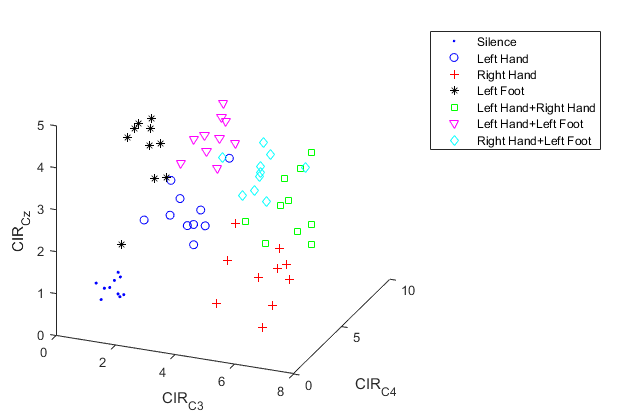

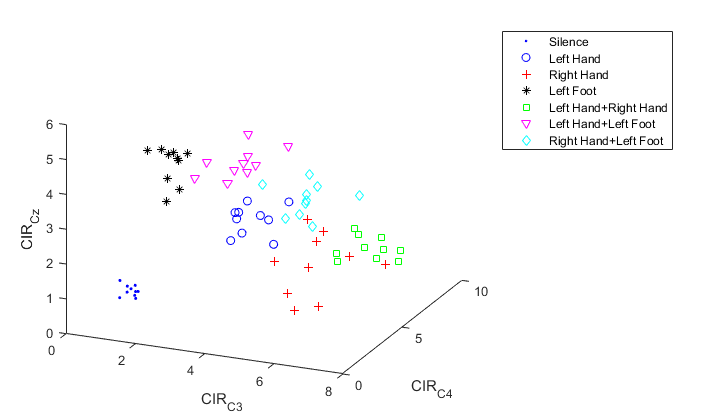

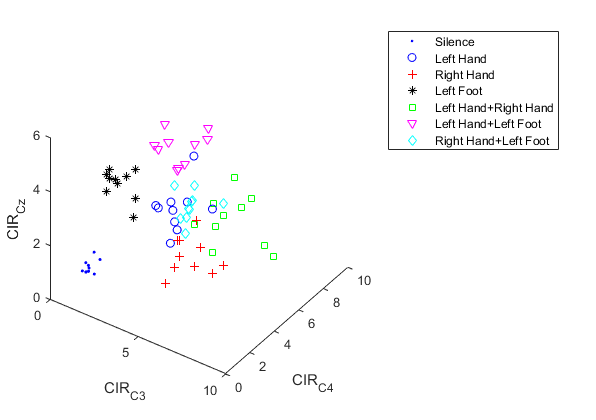

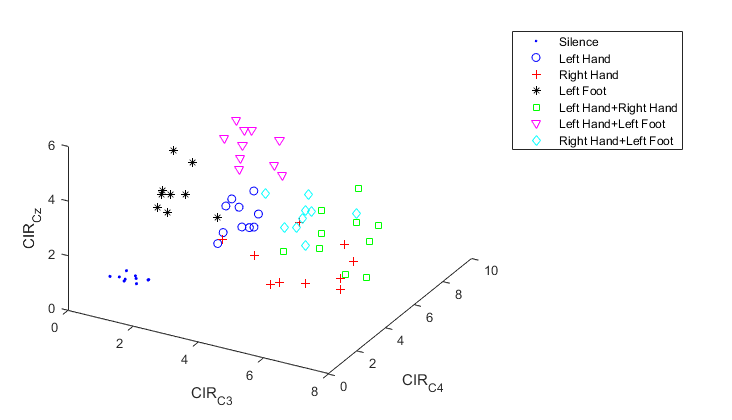

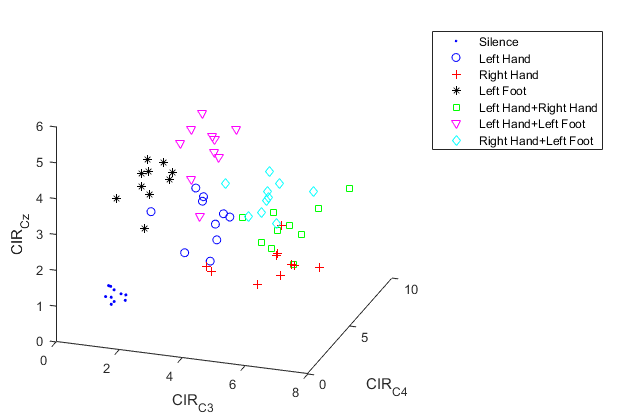

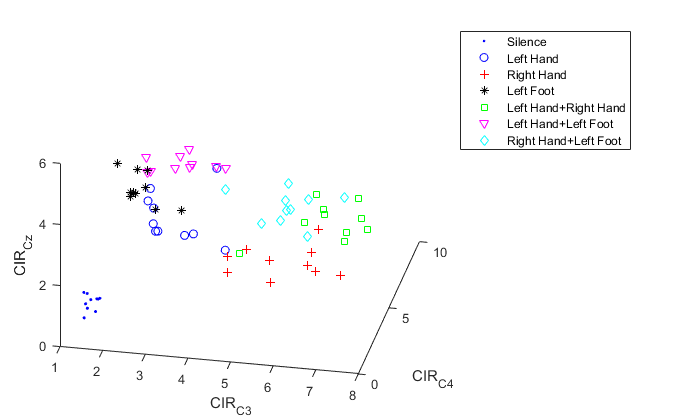

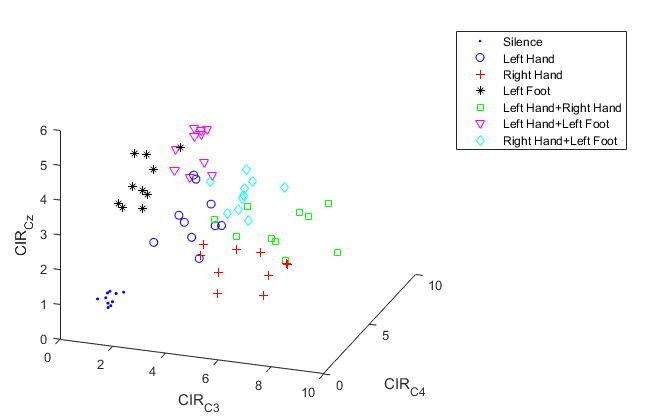

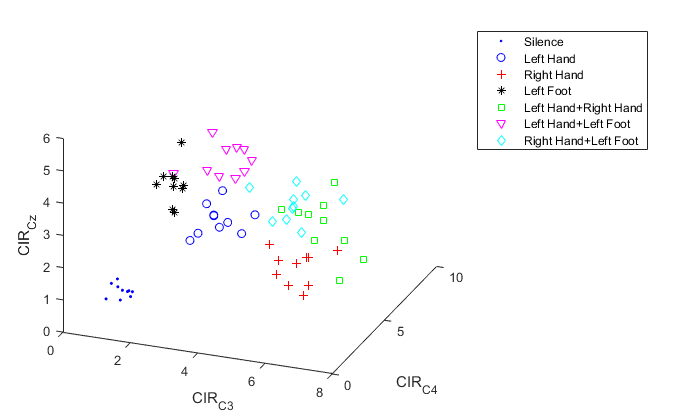

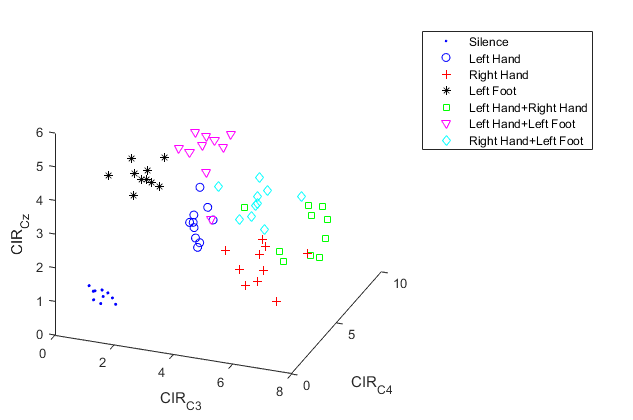

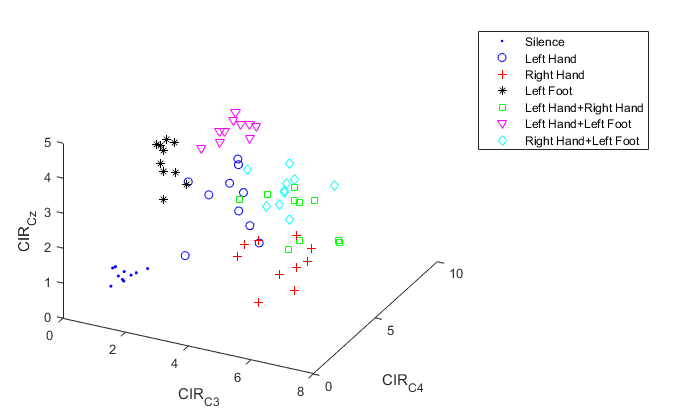

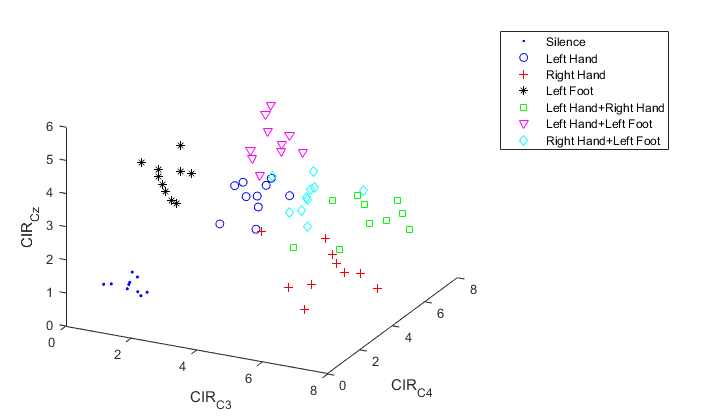

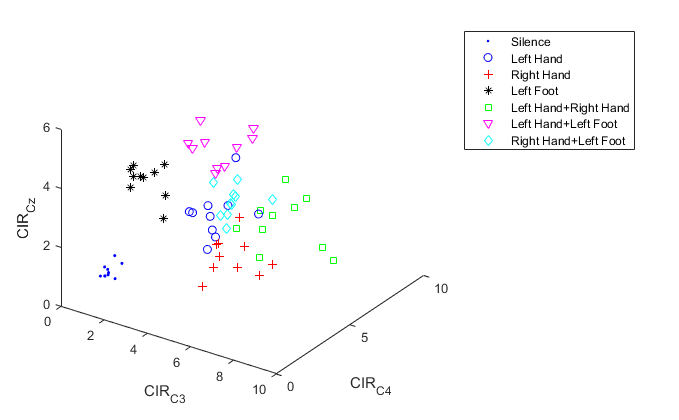

Supplement: Supplementary Materials — Figure S1: the CIR distribution of the 7 mental tasks in each individual subject. [file 6655430.f1.docx]
